# Molecular Dynamics Simulations to Investigate How PZM21 Affects the Conformational State of the μ-Opioid Receptor Upon Activation

**DOI:** 10.3390/ijms21134699

**Published:** 2020-07-01

**Authors:** Zhennan Zhao, Tingting Huang, Jiazhong Li

**Affiliations:** School of Pharmacy, Lanzhou University, Lanzhou 730000, China; zhaozhn18@lzu.edu.cn (Z.Z.); huangtt18@lzu.edu.cn (T.H.)

**Keywords:** G protein-biased agonists, µ-opioid receptor (MOR), morphine, PZM21, molecular dynamics, simulation

## Abstract

Opioid analgesics such as morphine have indispensable roles in analgesia. However, morphine use can elicit side effects such as respiratory depression and constipation. It has been reported that G protein-biased agonists as substitutes for classic opioid agonists can alleviate (or even eliminate) these side effects. The compounds PZM21 and TRV130 could be such alternatives. Nevertheless, there are controversies regarding the efficacy and G protein-biased ability of PZM21. To demonstrate a rationale for the reduced biasing agonism of PZM21 compared with that of TRV130 at the molecular level, we undertook a long-term molecular dynamics simulation of the μ-opioid receptor (MOR) upon the binding of three ligands: morphine, TRV130, and PZM21. We found that the delayed movement of the W293^6.48^ (Ballesteros–Weinstein numbering) side chain was a factor determining the dose-dependent agonism of PZM21. Differences in conformational changes of W318^7.35^, Y326^7.43^, and Y336^7.53^ in PZM21 and TRV130 explained the observed differences in bias between these ligands. The extent of water movements across the receptor channel was correlated with analgesic effects. Taken together, these data suggest that the observed differences in conformational changes of the studied MOR–ligand complexes point to the low-potency and lower bias effects of PZM21 compared with the other two ligands, and they lay the foundation for the development of G protein-biased agonists.

## 1. Introduction

Pain is a highly unpleasant physical sensation caused by illness or injury. It is a common symptom associated with many diseases. With increases in people’s wishes for having a good quality of life, the expectation of treating pain (especially cancer-related pain) is increasing.

Morphine [1] is used worldwide because of its strong analgesic activity. However, morphine can produce a series of toxic reactions and side effects: tolerance, constipation, respiratory depression, and addiction. The US Centers for Disease Control and Prevention (Atlanta, GA, USA) has announced that opioid abuse has become one of the most serious public health crises in the USA in the past decade. The number of poisoning incidents caused by opioid abuse is numerous. In 2015, more than 33,000 people in the USA died from an overdose of opioids [2]. In 2017, approximately 50,000 deaths were caused by opioid abuse. From 2001 to 2016, the total number of opioid-related deaths was >335,000 [3]. The World Health Organization claims that about 275 million people worldwide (5.6 per cent of the global population aged 15–64 years) used drugs at least once during 2016. Among them, there were about 34 million people who used opioids and about 19 million who used opiates [4]. However, >125 million US citizens are suffering from acute or chronic pain, and this number is even greater in the world.

Morphine targets the μ-opioid receptor (MOR), which is a member of the G protein-coupled receptor (GPCR) family. Originally, it was believed that GPCRs could activate only downstream G proteins. However, researchers have found that GPCRs not only activate G proteins, but also activate other signal transducers, such as β-arrestin and scaffold proteins [5]. Mice lacking *β-arrestin* have been shown to have lower resistance to morphine, an increased analgesic effect, and prolonged drug action compared with those from wild-type mice, and the desensitization effect is reduced [6,7,8]. These results indicate indirectly that side effects are associated with the β-arrestin pathway. It has not been demonstrated directly that the β-arrestin pathway mediates side effects such as respiratory depression in mice. However, opioid agonists are now recognized widely as activating the G-protein pathways to produce an analgesic effect, and that some side effects may be associated with activation of the β-arrestin pathway.

Developing opioid substitutes that are efficacious with few side effects is the best long-term solution for controlling the side effects mentioned above. Following this hypothesis, several G-protein-biased agonists have been developed in the 21st century. For example, using the natural product mitragynine (an indole-based alkaloid from *Mitragyna speciose*) and its analogs [9], Schmid et al. [10] postulated that SR17018, SR15099, and SR-15098 could be potential G protein-biased agonists. TRV130 (Oliceridine) [11,12,13] (Trevena, Chesterbrook, PA, USA) not only has good analgesic activity but also has G protein-biased properties, so it has been granted the designation of “breakthrough therapy” by the US Food and Drug Administration (FDA) and has completed phase III clinical trials. In November 2017, Trevena submitted a new drug application registration to the FDA.

A G protein-biased agonist reported recently is the compound PZM21. The latter has been screened using molecular docking and modified subsequently [14]. Manglik and colleagues showed that PZM21 has a strong analgesic effect (EC_50_ = 4.6 nM) and no morphine-like side effects. It has garnered considerable attention because of its potential medicinal value, and researchers have conducted in-depth research into PZM21. However, Hill et al. [15] demonstrated that PZM21 does not have a close association with a G protein-based pathway (EC_50_ = 110 nM) or β-arrestin pathway, and it is a dose-dependent analgesic and G protein-biased agonist.

**Figure 1:**
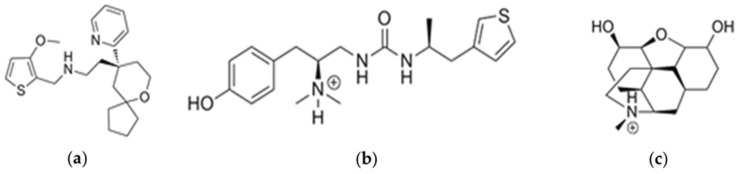
Chemical structures of the studied ligands: (**a**) TRV130, (**b**) PZM21, and (**c**) morphine.

We wished to clarify the effect of PZM21 in a theoretical fashion. We used an all-atomic molecular dynamics (MD) simulation to compare the movements of the non-biased agonist morphine and biased agonists TRV130 and compound PZM21. In this way, we investigated the conformational changes of the MOR influenced by the three ligands, respectively. The chemical structure of the three ligands is shown in Figure 1.

## 2. Results and Discussion

### 2.1. Molecular Docking Results of Each System

**Figure 2:**
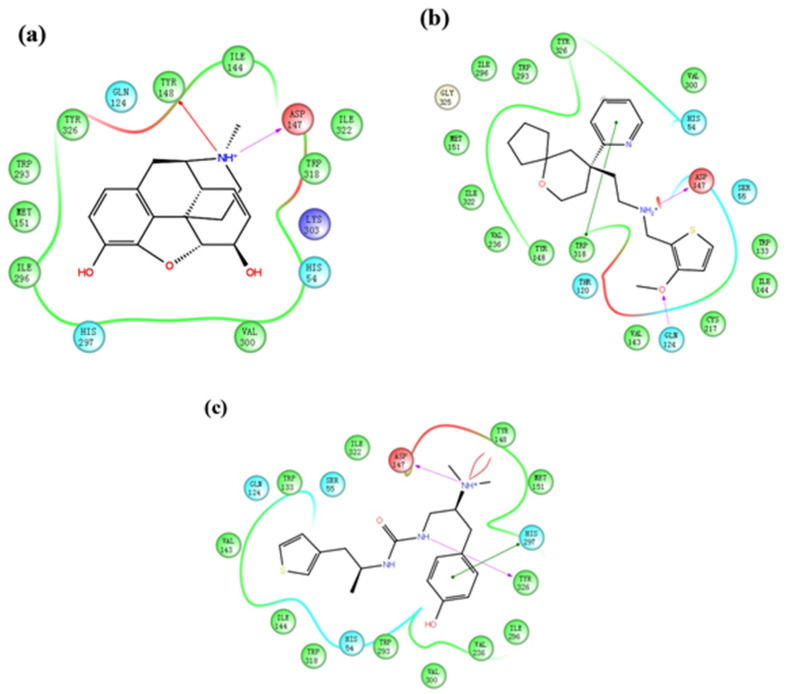
Molecular docking results for each system. (**a**) Morphine_docked, (**b**) TRV130_docked, (**c**) PZM21_docked.

The two-dimensional diagram of the interactions between a ligand and various protein residues are shown in Figure 2. The tertiary amine cations of morphine, TRV130, and PZM21 had a hydrogen bond with D147^3.32^ (Ballesteros–Weinstein numbering [16]), data that are consistent with results from previous studies [14,17]. An interaction between morphine and Y148^3.33^ was noted, and strong π–π stacking was present between the pyridine ring of TRV130 and W318^7.35^. PZM21 contains three nitrogen atoms, so there were two interactions in this system: (1) a hydrogen bond between PZM21 and Y326^7.43^, and (2) π–π stacking between the benzene ring and H297^6.52^. We used these molecular docking results as the initial structures for the next MD simulation.

### 2.2. Stability of Each MD Simulation System

**Figure 3:**
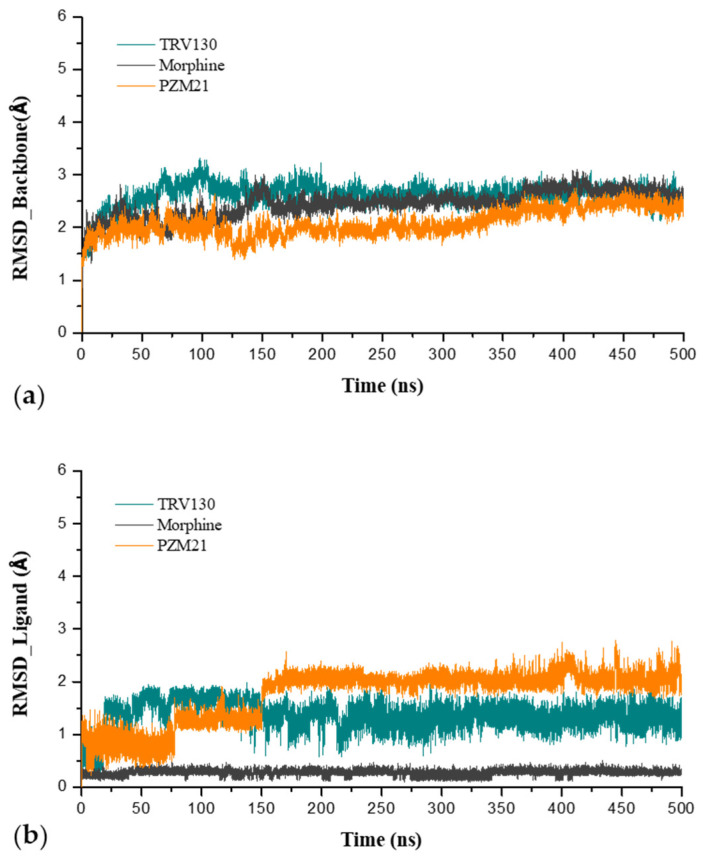
(**a**) Root-mean-square deviation (RMSD) of the protein backbone and (**b**) RMSD of the ligand.

We wished to monitor the stability of the system. Hence, after three 500 ns unbiased MD simulations, we calculated the RMSD of the protein backbone atoms and ligands (Figure 3). The protein volatility in TRV130_sys was relatively large and eventually stabilized at approximately 2.7 Å, followed by Morphine_sys at approximately 2.6 Å and PZM21_sys at approximately 2.3 Å. The results suggested that TRV130_sys had a large fluctuation in amino acid changes compared with those of Morphine_sys and PZM21_sys, which affected the conformational changes of the protein to a large extent. However, for the ligands, the fluctuations of the three systems were ranked as PZM21_sys (2.0 Å), TRV130_sys (1.3 Å), and Morphine_sys (0.3 Å). These fluctuations occurred because the molecular structure of PZM21 has a longer side chain, which is more flexible and prone to fluctuation. The molecular structure of morphine contains five rings of a morphinan core that is relatively rigid and does not undergo fluctuation readily. Overall, the three systems eventually reached a relatively stable state after 400 ns, so we used the last 100 ns of the MD simulation for subsequent analyses.

### 2.3. Residue D147^3.32^ and H297^6.52^ in the Ligand-Binding Pocket

**Figure 4:**
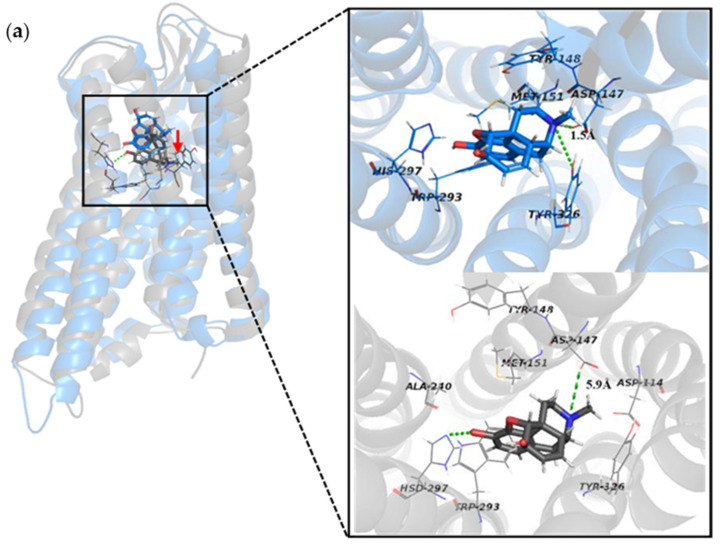

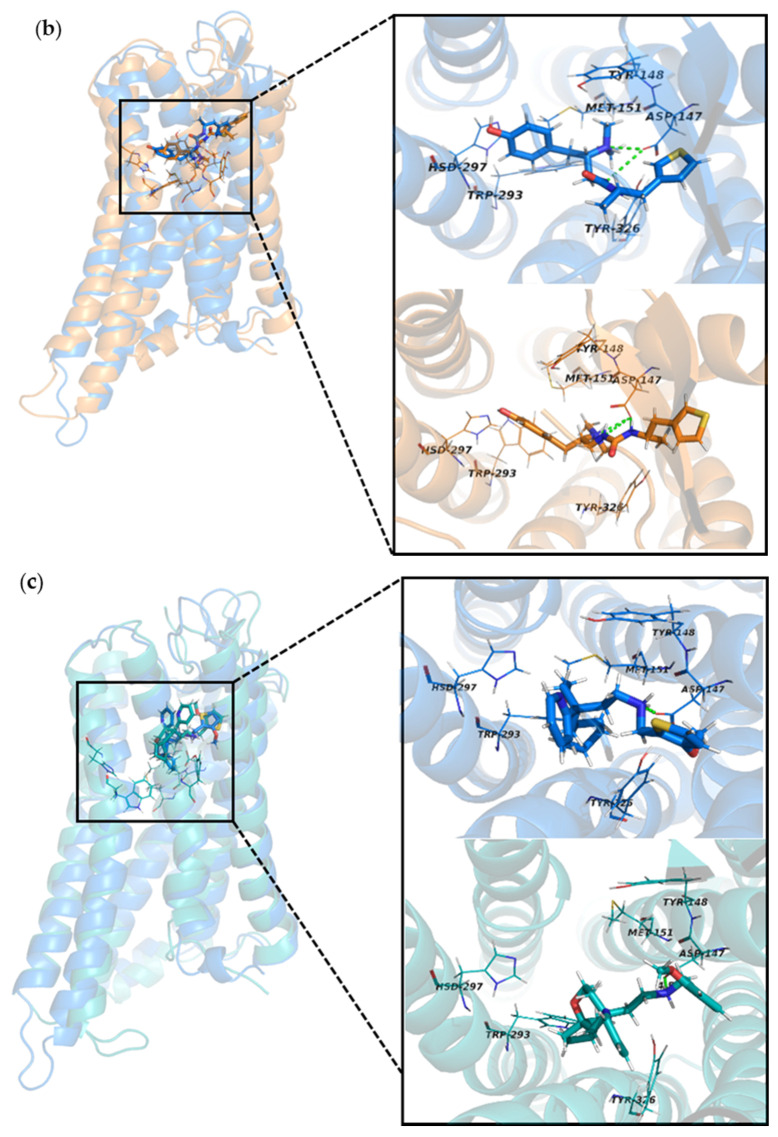
Superposition of the docked structure and simulated structure of the three systems and an image of the ligands and partial residues in the binding pocket. (**a**) Docked complex/blue vs. simulated complex/gray in Morphine_sys. (**b**) Docked complex/blue vs. simulated complex/orange in PZM21_sys. (**c**) Docked complex/blue vs. simulated complex/green in TRV130_sys.

An interaction between the morphinan tertiary amine cation of the ligand and the polar residue D147^3.32^ was found in the crystal structure of the activated MOR bound to the agonist BU72 and the crystal structure of the inactive MOR [18] bound to the antagonist β-funaltrexamine. Based on this interaction, compound PZM21 was discovered by means of molecular docking [14]. H297^6.52^ (also located in the active pocket) formed a hydrogen bond with the phenolic hydroxyl group of BU72 or generated a water-mediated network of hydrogen bonds. In the activated pocket, this hydrogen bond network extended to Y148^3.33^ [19]. To estimate the affinity between the ligands morphine, PZM21, TRV130, and protein, we calculated the proportion of these hydrogen bonds in the entire MD simulation, including D147^3.32^ with ligands and water (Table 1). At least one hydrogen bond was formed between D147^3.32^ and PZM21/TRV130, so both of them could bind closely to the MOR. The hydrogen bond between D147^3.32^ and morphine accounted for only 6.58%, and the hydrogen bond broke rapidly during the MD simulation. To confirm this result intuitively, we aligned the docked complex and simulated complex (Figure 4). In the docked result, the distance between morphine and D147^3.32^ was 1.5 Å, and after a period of dynamic simulation, the distance was increased to 5.9 Å. This result was due to the fact that morphine penetrates deep into the pocket and forms a more stable hydrogen bond with H297^6.52^. Conversely, PZM21 and TRV130 did not move deep inside the pocket during the simulation. The distance between D147^3.32^ and PZM21/TRV130 was kept at a short range, and the hydrogen bond could continue to be formed. Moreover, PZM21 also formed a stable hydrogen bond with H297^6.52^. These results indicated that these ligands were targeted to the MOR.

### 2.4. Residues W293^6.48^, Y326^7.43^, W318^7.35^, and Y336^7.53^ Affect Receptor Function

Studies have shown that W6.48 is an “activation switch” [20] that activates “water channels”; W6.48 has an important effect on activation of opioid receptors. Conformational changes of W6.48 in the δ-opioid receptor regulate the conformation of transmembrane 5 (TM5) and TM6, and they transmit signals to the intracellular loop region to upregulate the selectivity of the G protein [21]. Hence, we focused on study of the key residue W6.48 and calculated the change in the torsion angle of the W293^6.48^ side chain.

**Figure 5:**
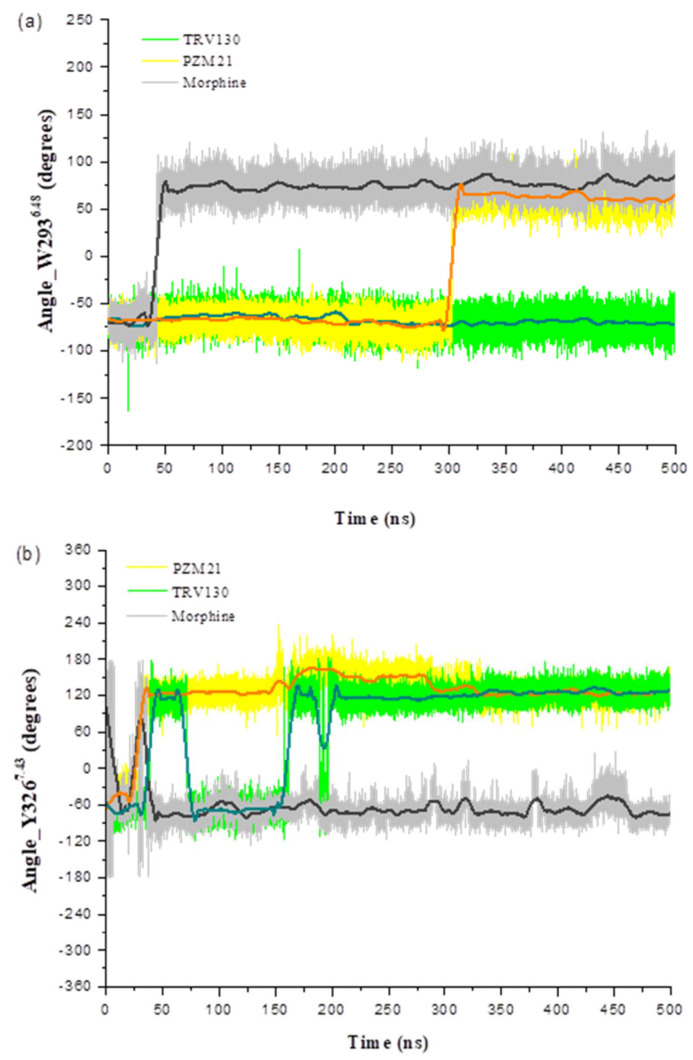
Change in torsion angle of residues (**a**) W293^6.48^ and (**b**) Y326^7.43^ in three systems.

**Figure 6:**
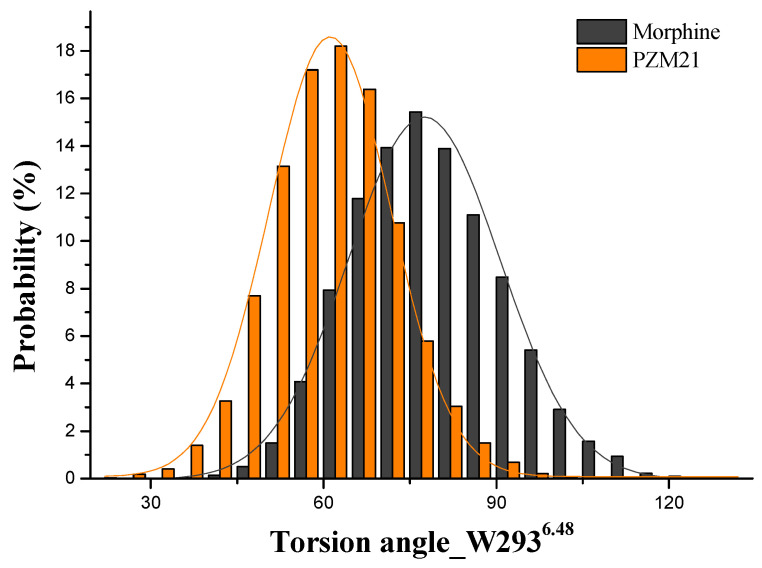
Distribution of the torsion angle of residue W293^6.48^ in Morphine_sys and PZM21_sys.

The W293^6.48^ side chains in PZM21_sys and Morphine_sys were finally reversed (Figure 5a). That reversal occurred in Morphine_sys at 50 ns, but it occurred in PZM21_sys at 300 ns. A statistical histogram for the W293^6.48^ frequency (Figure 6) shows that the reversed angle in Morphine_sys was stable at approximately 76°, whereas in PZM21_sys it was stable at approximately 62°. We deduced that the difference in variation of the W293^6.48^ side chain between Morphine_sys and PZM21_sys might be an important factor leading to a higher dose of PZM21 than morphine to achieve similar agonist ability. This conclusion is consistent with the study of Hill et al. [15]. The torsion angle of the W293^6.48^ side chain of TRV130_sys barely changed throughout the simulation. Overlap of the docking structure and simulated structure revealed that a translation to the binding pocket occurred in W293^6.48^, so TRV130 may differ from other ligands in terms of activating the MOR.

**Figure 7:**
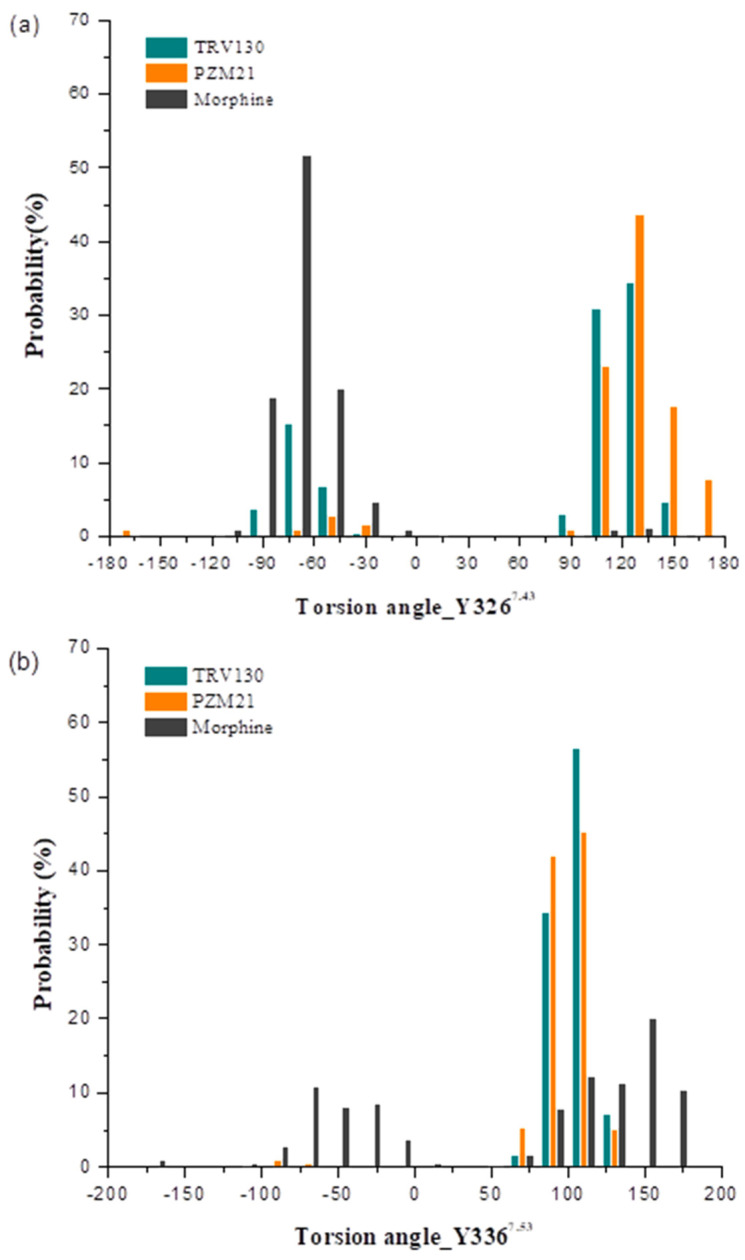
Distribution of the torsion angle of residues (**a**) Y3267^.43^ and (**b**) Y336^7.53^ in three systems.

**Figure 8:**
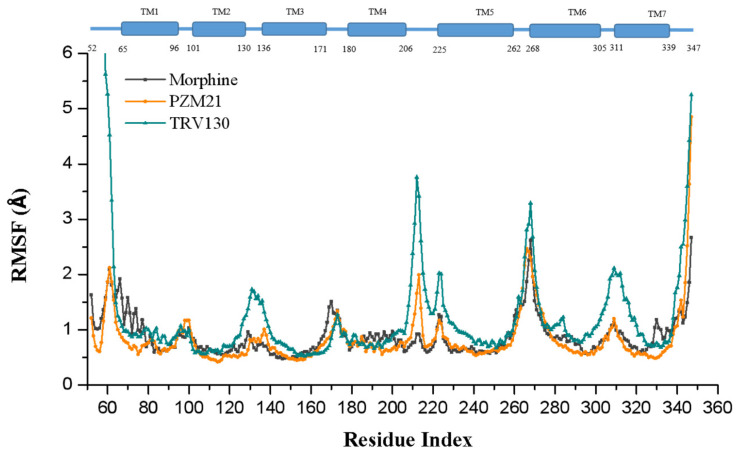
The root-mean-square fluctuation (RMSF) of the MOR backbone in three systems.

Moreover, Y326^7.43^ and W318^7.35^ also had a certain effect on the bias of the agonist. In the Y7.43F mutant system, [D-Ala2,N-MePhe4,Gly-ol]-enkephalin (DAMGO) completely loses the recruitment of β-arrestin-2, but in the W7.35A mutant system, it increases the relative activity of β-arrestin-2 [22]. We calculated the change in torsion angle of the Y326^7.43^ side chain and root-mean-square fluctuation (RMSF) of residues. Y326^7.43^ in Morphine_sys had almost no turning, and the deflection angle was stable at about −75° (Figure 5b). The Y326^7.43^ side chain in PZM21_sys and TRV130_sys was deflected, and the deflection angle was stable at approximately 125°. The Y326^7.43^ side chain of TRV130_sys fluctuated from −100° to 150°, and in PZM21_sys, it fluctuated from 100° to 175° (Figure 7). The Y326^7.43^ side chain of TRV130_sys was more active than that in PZM21_sys. Figure 8 exhibits the RMSF of the MOR backbone in three systems, and it shows that W318^7.35^ in TRV130_sys fluctuated the most, but it maintained similar stability in Morphine_sys and PZM21_sys. The MD simulation of Cheng et al. [23] showed that the stability of Y326^7.43^ and W318^7.35^ was related to the bias of the agonist. Hence, the changes of these residues in our simulation results confirmed that PZM21 and TRV130 had a certain degree of bias. However, the bias of PZM21 was not as strong as that of TRV130, and these data are in accordance with the experimental results of Hill et al. [15]. Furthermore, Y336^7.53^ in TRV130_sys and PZM21_sys had similar fluctuations of the side-chain torsion angle ranging from approximately 75° to 125°, but TRV130_sys was more stable than PZM21_sys. Fluctuations of the side-chain torsion angle in Morphine_sys ranged from −80° to 175°, and these fluctuations were in a wide range. Y336^7.53^ was located in the G protein-binding region, and the distribution of the side-chain torsion angle indicated that the mechanism of the three ligands affecting the downstream pathway of MOR activation was different.

### 2.5. The Water Channel Associated with Activation

**Figure 9:**
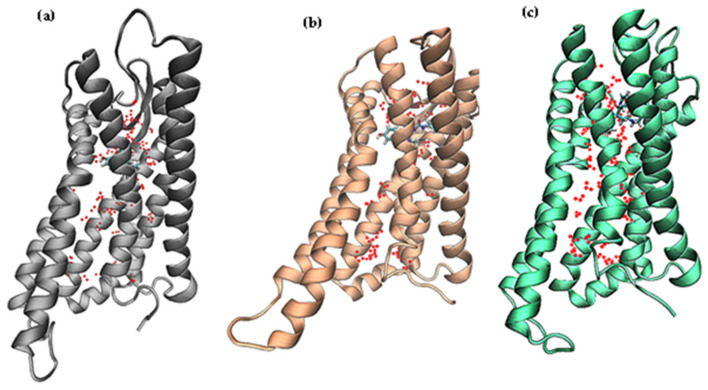
Image showing the equilibrated structure of the “water channel” in (**a**) Morphine_sys, (**b**) PZM21_sys, and (**c**) TRV130_sys. Each red dot represents a water molecule.

**Figure 10:**
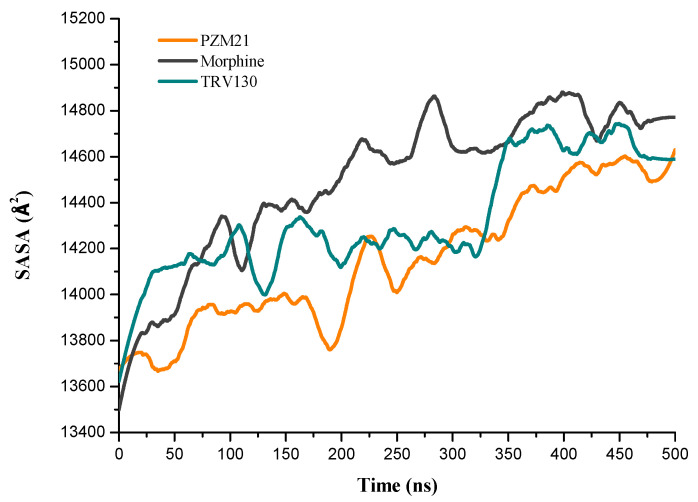
Solvent-accessible surface area (SASA) of the transmembrane region of the MOR in different systems with time.

Studies have shown that a ligand located in the orthosteric site can mediate the entry of water molecules into the G protein-coupled receptor to cause receptor activation [20,24]. An image of the equilibrated conformations of the three systems (Figure 9) revealed that the water molecules entering the transmembrane (TM) region were the most in Morphine_sys, followed by TRV130_sys, and finally PZM21_sys. These data were validated when the statistics of the hydrogen-bond ratio were calculated (Table 1). Compared with PZM21_sys, TRV130_sys and Morphine_sys had more hydrogen bonds between the residue of the binding pocket and water. The solvent-accessible surface area (SASA) of the MOR TM region can reflect the activation effect of the ligand on the receptor. Thus, we calculated the SASA of the MOR TM region in the three systems (Figure 10). The SASA had an upward trend in the three systems. Eventually, Morphine_sys reached 14.800 Å^2^, TRV130_sys reached 14.600 Å^2^, and PZM21_sys reached 14.400 Å^2^. These results were in accordance with the data shown in Figure 9, and indicated that the ability to activate the MOR followed the order of morphine, TRV130 and PZM21.

### 2.6. Flexibility and Conformational Change in the Loop Area

The RMSF of the three systems is shown in Figure 8, and the overall fluctuation was similar. The less flexible parts were the relatively rigid TM regions, and the seven regions with lower RMSF values can be seen in Figure 8. Seven-segment TM helices were represented, and the residue numbers were 65–96 (TM1); 101–130 (TM2); 136–171 (TM3); 180–106 (TM4); 225–262 (TM5); 268–305 (TM6); and 311–339 (TM7). The flexible regions with large RMSF values corresponded to the intracellular and extracellular loop regions of the MOR: 97–100 (Intracellular 1, I1), 131–135 (Extracellular 1, E1), 172–179 (Intracellular 2, I2), 207–224 (Extracellular 2, E2), 263–267 (Intracellular 3, I3) and 306–310 (Extracellular 3, E3), respectively. 52Gly and 347Phe, which locate the N-terminus and C-terminus of the receptor, respectively, had greater flexibility and higher RMSF values compared with other regions. In terms of overall similarity, there were subtle differences in the different systems due to the different ligands. Morphine_sys and PZM21_sys had similar RMSF distributions and similar dynamic characteristics, but the RMSF of TRV130_sys was larger than that of the other two systems, especially in the E1, E2, I3, and E3 regions. These dynamic differences can elicit different biologic effects by modulating the interaction between the receptor and a downstream signal-transduction protein such as a G protein or β-arrestin. Studies have shown that E2 and E3 have important roles in G-protein selectivity [5], and that I3 has a hydrophobic interaction with Gi or a polar interaction with the sixth β-sheet of Gα in the MOR. Therefore, our simulation results showed that TRV130, morphine, and PZM21 had different effects on the MOR, whereas PZM21 exhibited similar movements to that of morphine in some respects.

**Figure 11:**
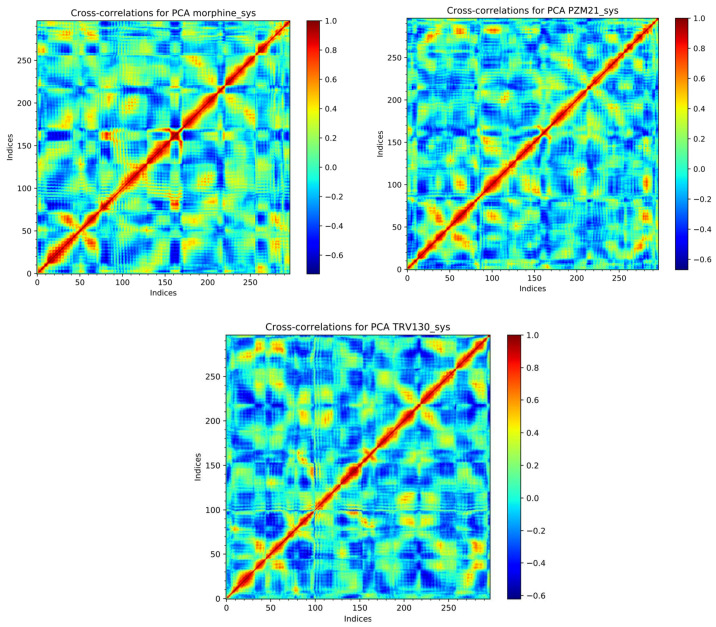
Dynamic cross-correlation matrix diagram for each system.

In order to better understand the effects of morphine, PZM21, and TRV130 on the conformational state of MOR, we calculated the dynamic cross-correlation matrix (DCCM) of the C_α_ atom of the receptor throughout the entire simulated trajectory (Figure 11). The red and yellow parts were called the “positive regions”, and they represented positive correlations between the residues (i.e., the dynamic movements of the residues were in the same direction). The blue portion was called the “negative region” and represented strong negative correlations between the residues (i.e., the movements of the residues were opposite). We noted few negative regions in Morphine_sys containing the non-biased agonist, and many negative regions in the system in which the bias agonist TRV130 was present. A negative-correlation motion of PZM21_sys was between Morphine_sys and TRV130_sys. These results suggested that (1) morphine, PZM21, and TRV130 had different effects on the local dynamics and conformation of the receptor protein; (2) the biased agonist could cause more negative correlation movement of the receptor protein compared with unbiased agonist morphine.

## 3. Materials and Methods

### 3.1. Preparation and Molecular Docking

The crystal structure of the activated MOR (PDB: 5C1M [19]) was downloaded from the Protein Data Bank (www.rcsb.org). The crystal structure bound with the agonist BU72 contains a nanobody (Nb39) to stabilize the MOR structure. To carry out MD simulations on the wild-type MOR, we removed Nb39 and mutated the non-standard amino acid YCM back to cysteine. We left only the ligand BU72 at the orthosteric site as a binding site for the subsequent molecular-docking study. Then, we complemented missing amino acids and loop fragments in the crystal. The three-dimensional structures of morphine, TRV130, and PZM21 were constructed in Discovery Studio 2.5 [25]. Ligands were prepared by the “Prepare Ligands” module to obtain the lowest energy and more stable ligand structure.

First, we used Maestro v10.1 (Schrödinger, New York, NY, USA) for docking studies. Initially, “Protein Preparation Wizard” was used to examine and optimize the protein. The “PROPKA” tool was employed to adjust the protein to a physiologic pH = 7 environment. Hydrogen atoms were added to the structure, and a Monte Carlo multiple minimum conformation search was done under the OPLS_2005 force field. The “Ligprep” tool is used to neutralize ligands and determine chiralities from 3D structure. Second, the ligand BU72 in the protein structure was set to the center of the binding-site box, the radius of which was 15 Å. Finally, the three ligands were docked into the receptor using Glide 5.8 XP [26]. The conformation results were arranged in order of scoring, and a reasonable (i.e., the docking score was high and the key interaction between the ligand and receptor was present) complex conformation was selected as the initial structure. To ensure the reliability of our docking results, we used the same method to dock BU72 in the crystal structure to the receptor with a root-mean-square deviation (RMSD) of 0.496 Å. Therefore, our docking results were credible.

### 3.2. Construction of the Simulation System

**Figure 12:**
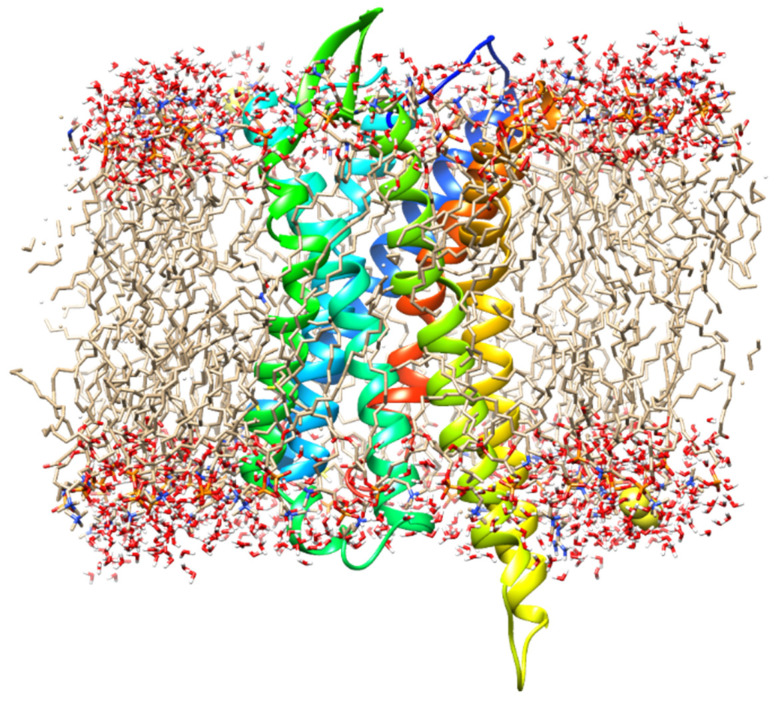
Sketch map of the simulation system including the μ-opioid receptor (MOR), lipid membranes, and water molecules. For clearer display, some water molecules and the ligand are hidden.

The simulation system consisted of 1-palmitoyl-2-oleoylsn-glycero-3-phosphocholine (POPC), the MOR, and ligands (Figure 12). The membrane was composed of two layers of POPC lipid molecules, each of which had the same initial conformation (the hydrophobic tail of the molecule was straight).

First, the complexes of the MOR and ligand were inserted into a lipid membrane composed of 89 POPC lipid molecules, and the major axis of the MOR was made substantially perpendicular to the membrane surface. Second, some of the amino acids in the protein were charged, so we utilized NaCl solution (0.15 M) to maintain the neutrality of the system. Finally, the system was solvated by adding a certain number of water molecules of the TIP3P model throughout the system. Each simulation system had a size of about 8 × 8 × 10 nm^3^ and contained approximately 40.000 atoms. Construction of the entire simulation system was completed using the CHARMM-GUI Internet server [27,28,29] (www.charmm-gui.org), and the simulation details are shown in Table 2.

### 3.3. MD Simulations

The conduction of MD simulations were proceeded with software GROMACS 5.1.4. (manual.gromacs.org/). The CHARMM36 force field [30,31] was described for the protein and membrane. The Charmm General Force Field (CGenFF) via the Paramchem Internet site was described for parametrized ligands. van der Waals interactions were calculated using a double cutoff radius of 10–12 Å. The long-range electrostatic force was calculated using the Particle–Mesh–Ewald method [32]. Using periodic boundary conditions, hydrogen atoms were constrained by the LINear Constraint Solver (LINCS) algorithm [33]. The time step was 2 fs.

First, 5000-step energy optimization was undertaken by the steepest-descent method to avoid the local irrationality of the system. Second, due to the particularity of the membrane system, a special equilibrium scheme was adopted. The Bernendson method [34] is used to control the temperature at 310K while maintaining a constant pressure of 1 atm for a conical ensemble (NVT) and isobaric–isothermal ensemble (NPT) ensemble. In this process, the backbone and side chains of the protein and ligands are given a different constraint potential in each step. Finally, all the constraints are removed, and the system is released completely for 1 ns pre-equalization. The equilibration time and the constraint force parameters are shown in Table 3. The temperature and pressure of this process were controlled by the Nosé–Hoover method [35] and Parrinello–Rahman method [36], respectively. Finally, each system was subjected to a MD simulation of 500 ns.

## 4. Conclusions

The development of safe and efficacious analgesic agents with few side effects is an urgent need. The common feature of such analgesics is that they are biased toward a G-protein pathway. The biased properties of compound PZM21 and its analgesic activity are controversial.

We investigated the effects of the biased agonist TRV130, unbiased agonist morphine, and compound PZM21 on the MOR by means of MD simulations. Our results enabled five main conclusions to be drawn.

First, morphine, PZM21, and TRV130 formed stable hydrogen bonds with residues D147^3.32^ and H297^6.52^ to aid ligand binding to the MOR. All ligands targeted the MOR.

Second, we analyzed some key residues associated with receptor selectivity and noted two main trends. The first trend we noticed was that the side chain of W293^6.48^ was reversed in PZM21_sys and Morphine_sys, but it reversed slowly in PZM21_sys. This may be an important factor why PZM21 is a dose-dependent agonist. The second trend we identified was that the Y326^7.43^ side chain rotated to a certain degree in PZM21_sys and TRV130_sys, but that it had a larger fluctuation range and was more active in TRV130_sys. Y336^7.53^ in PZM21_sys was more active and W318^7.35^ in TRV130_sys was more active compared with PZM21_sys. Rotation of the side chains in Y326^7.43^ and Y336^7.53^ was similar but slightly different in TRV130_sys and PZM21_sys. These data may imply that they were biased, but PZM21 was less biased than TRV130.

Third, the extent of openness of the water channel affected the analgesic activity of the ligand. The SASA of Morphine_sys was the largest, followed by TRV130, and finally PZM21. Therefore, the analgesic activity of PZM21 merits additional study.

Fourth, by analyzing the flexibility of the loop region, the fluctuations of Morphine_sys and PZM21_sys in the E1, E2, I3, and E3 regions were significantly smaller than those of TRV130_sys. In addition, PZM21 exhibited similar dynamic properties to those of morphine in these regions.

Finally, DCCM analyses revealed that negative correlation movements between residues in the biased agonist system were more common compared with the unbiased agonist system, whereas the negative correlation between the residues of PZM21_sys was between Morphine_sys and TRV130_sys.

In summary, our simulation work indicated that PZM21 did not appear to have an obvious advantage such as that of TRV130 in terms of analgesia or fewer side effects. From its dynamic behavior, PZM21 was a low-potency analgesic and a low-potential biased agonist. These data are in good agreement with the experimental results of Hill et al. [15]. We also summarized some movements related specifically to biased agonists, which lay the foundation for the design of morphine substitutes with fewer side effects (i.e., G protein-biased agonists).

## Figures and Tables

**Table 1:** Occupancy of hydrogen bonds between D3.32 and water, and D3.32 and ligands, in each system. Data were calculated by Groningen Machine Chemical Simulations (GROMACS). Determination of hydrogen bonding is based on the conditions of α ≤ 30° and r ≤ 0.35 nm.

H-bond Number	D3.32_H_2_O			D3.32_Ligand		
	Morphine (%)	PZM21 (%)	TRV130 (%)	Morphine (%)	PZM21 (%)	TRV130 (%)
0	− ^a^	0.04%	0.23%	93.42%	−	17.43
1	0.03	3.40	1.18	5.62	1.18	65.44
2	0.35	51.47	1.85	0.96	41.68	17.14
3	3.78	35.85	3.67	−	40.03	−
4	31.79	7.49	20.90	−	16.09	−
5	47.21	1.58	52.27	−	1.02	−
6	14.19	0.16	15.36	−	−	−
7	2.43	0.01	4.00	−	−	−
8	0.20	−	0.52	−	−	−
9	0.02	−	0.02	−	−	−

^a^ The symbol “−” denotes no related interaction with a hydrogen bond.

**Table 2:** Simulations systems used in this study.

Name of System	Number						Simulation Time/ns
	POPC	TIP3P	Na^+^	Cl^−^	Ligand	Receptor	
Morphine_MOR	89	7764	31	45	Morphine	MOR	500
PZM21_MOR	89	7765	31	45	PZM21	MOR	500
TRV130_MOR	89	7003	18	32	TRV130	MOR	500

**Table 3:** Equilibration time and constraint force parameters of each step. NPT: isobaric–isothermal ensemble, NVT: conical ensemble.

Step	Ensemble	Time Steps /fs	Equilibration Time/ps	Force Constant/KJ/mol·nm^−2^		
				Protein Backbone	Protein Side Chain	Ligand
1	NVT	1	25	4000	2000	4000
2	NVT	1	25	2000	1000	2000
3	NPT	1	25	1000	500	1000
4	NPT	2	100	500	200	500
5	NPT	2	100	200	50	200
6	NPT	2	100	50	0	50
7	Pre-Production	2	1000	0	0	0
